# CT Texture Analysis—Correlations With Histopathology Parameters in Head and Neck Squamous Cell Carcinomas

**DOI:** 10.3389/fonc.2019.00444

**Published:** 2019-05-28

**Authors:** Hans-Jonas Meyer, Gordian Hamerla, Anne Kathrin Höhn, Alexey Surov

**Affiliations:** ^1^Department of Diagnostic and Interventional Radiology, University of Leipzig, Leipzig, Germany; ^2^Department of Pathology, University of Leipzig, Leipzig, Germany

**Keywords:** CT, texture analysis, HNSCC, Hif1-alpha, Ki67

## Abstract

**Introduction:** Texture analysis is an emergent imaging technique to quantify heterogeneity in radiological images. It is still unclear whether this technique is capable to reflect tumor microstructure. The present study sought to correlate histopathology parameters with texture features derived from contrast-enhanced CT images in head and neck squamous cell carcinomas (HNSCC).

**Materials and Methods:** Twenty-eight patients with histopathological proven HNSCC were retrospectively analyzed. In every case EGFR, VEGF, Hif1-alpha, Ki67, p53 expression derived from immunhistochemical specimen were semiautomatically calculated. Furthermore, mean cell count was estimated. Texture analysis was performed on contrast-enhanced CT images as a whole lesion measurement. Spearman's correlation analysis was performed, adjusted with Benjamini-Hochberg correction for multiple tests.

**Results:** Several texture features correlated with histopathological parameters. After correction only CT texture joint entropy and CT entropy correlation with Hif1-alpha expression remained statistically significant (*ρ* = −0.60 and *ρ* = −0.50, respectively).

**Conclusions:** CT texture joint entropy and CT entropy were associated with Hif1-alpha expression in HNSCC and might be able to reflect hypoxic areas in this entity.

## Introduction

Head and neck squamous cell carcinoma (HNSCC) is one of the most frequent malignancies and has an overall poor prognosis with a 5-year survival rate of 50% (1). Imaging modalities play an important role in tumor diagnosis and treatment response in daily clinical routine (2).

Nowadays, imaging modalities might not only provide information about tumor localization and possible metastatic sites but also provide information regarding tumor microstructure (3–5).

This can be provided with modern imaging analyses, such as texture analysis on routinely acquired images, like computed tomography (CT) or conventional MRI sequences (5–8). This can be important because CT and conventional MR sequences are available in all clinical centers and thus this technique can be easily used in clinical routine.

Furthermore, texture analysis can display qualitative and quantitative assessment of tumor heterogeneity by analyzing the distribution and relationships of voxel gray levels in images (9). Various different analysis methods have been described in the literature. The principle method, referred to as first order statistics, reflects the gray-level frequency distribution from a histogram involving every pixel intensity of the tumor. Frequently used parameters include standard deviation, skewness, kurtosis, entropy. Second order statistics use a run-length matrix and can objectify texture heterogeneity of an image in a specific direction. The gray-level co-occurrence matrix is another second order technique that describes how often a pixel with a specific value pairs in a specified spatial range of an image (9).

There are still few studies elucidating the associations between texture features and underlying histopathology, despite its recent publicity in radiology and especially oncologic imaging. Presumably, the heterogeneity information provided by texture analysis might also correlate with the heterogeneity in tumors on a histological level, and, thus, might be associated with cellularity, vessel density, or other tumor features such as proliferation index or hypoxic areas.

Some previous studies confirmed these assumptions. For example, it was shown that CT texture features correlated with TNM stages in gastric cancer, indicating that nodal positive and tumors with higher T-stages have higher levels of heterogeneity related features like entropy (10). Similar results were also reported for esophageal cancer (11)_._ Furthermore, several texture features could discriminate between high and low grade lung cancers (8, 12).

There are several immunhistochemical features in HNSCC, which are of clinical relevance. These features can reflect different crucial tumor aspects. There are features, which are angiogenesis related, like vascular endothelial growth factor (VEGF), hypoxic related, like hypoxia-inducible factor-1-alpha (Hif1-alpha), proliferation related (Ki67), the tumor suppressor gene p53, oncogenic features, such as epidermal growth factor (EGFR). All have been identified to predict prognosis and treatment response in these patients (13–16). Presumably, prediction of histopathology by imaging might be crucial in clinical routine for treatment response evaluation or novel prognosis related imaging biomarkers.

The aim of the present study was to elucidate possible associations between CT texture analysis parameters and histopathology in HNSCC.

## Materials and Methods

This retrospective study was approved by the institutional review board (Ethic committee of the university of Leipzig, study codes 180-2007, 201-10-12072010, and 341-15-05102015). All methods were performed in accordance with the relevant guidelines and regulations. All patients gave their written informed consent.

### Patients

Overall, 28 patients with primary HNSCC of different localizations were involved in the study. There were 7 (25%) women and 21 (75%) men with a mean age of 56.1 ± 9.8 years, range 33–77 years. The tumors were localized in the oral cavity (*n* = 18, 64.3%), followed by hypopharynx (*n* = 6, 21.4%), and larynx (*n* = 4, 14.3%). In most cases, high grade lesions (G3, *n* = 17, 60.7%) were diagnosed. Most frequently, the identified lesions were staged as T3 and T4 tumors with additional nodal metastases.

### CT

CT images was performed using a Biograph 16 PET/CT scanner (Siemens Medical Solutions, Erlangen, Germany). CT scan was performed after intravenous injection of 120 ml contrast agent (Imeron 300, Bracco Imaging, Constance, Germany) with a collimation of 16 × 0.75 mm, a tube voltage of 120 kVp and the use of angular and longitudinal dose modulation (CARE Dose4D®, Siemens Medical Solutions, Erlangen, Germany).

### Imaging Analysis

**Figure 1:**
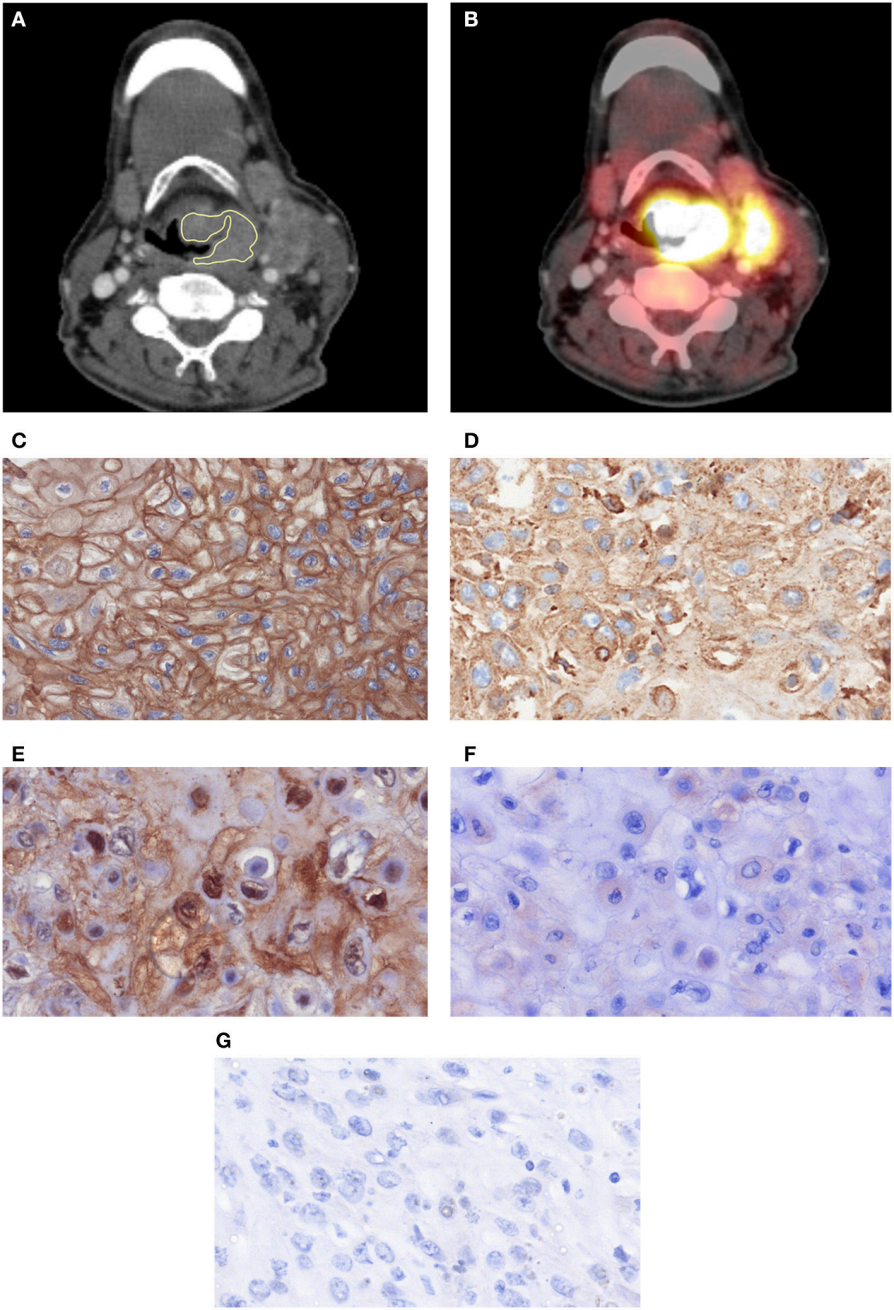
One case with a histopathologically confirmed HNSCC: postcontrast CT **(A)** in axial slices with placed ROIs and the corresponding PET overlay **(B)**. A cervical lymph node metastasis is also displayed. **(C)** EGFR-stained specimen. The stained area is 99,841 μm^2^. **(D)** Her 2-stained specimen. The stained area is 79,667 μm^2^. **(E)** Hif1-alpha stained specimen. The stained area is 14,896 μm^2^. **(F)** VEGF stained specimen. The stained area is 373 μm^2^. **(G)** P53 stained specimen. No immunreaction is seen.

For every lesion, CT images in uncompressed DICOM format were analyzed using MeVisLab (MeVis Medical Solutions AG, Bremen, Germany) on a standard Windows operating system. On the next step, on the saved maps, a volume of interest was drawn at tumor boundary using all slices (whole lesion measurement). Figures 1A,B shows a patient of the patient sample. During segmentation care was taken to include only voxels that resemble vital tumor tissue, avoiding surrounded vessels or cysts as well as partial volume effects. All measures were performed by two authors (GH, 2 years of radiological experience and AS, 15 years of radiological experience) in consensus. The extraction of texture features was carried out using validated software (17, 18), resulting in 95 features per case.

### Histopathological Findings

In all cases the diagnosis was confirmed histopathologically by tumor biopsy. The biopsy specimens were deparaffinized, rehydrated and cut into 5 μm slices. Thereafter, the histological slices were stained by epidermal growth factor receptor (EGFR, EMERGO Europe, clone 111.6, dilution 1:30), vascular endothelial growth factor (VEGF, EMERGO Europe, clone VG1, dilution 1:20), tumor suppressor protein p53 (DakoCytomation, Glostrup, Denmark; clone DO-7, dilution 1:100), Hif1-alpha (Biocare Medical, 60 Berry Dr. Pacheco, CA 94553; clone EP1215Y, dilution 1:100), according to previous descriptions (13–16).

Thereafter, all stained specimens were digitalized by using the Pannoramic microscope scanner (Pannoramic SCAN, 3DHISTECH Ltd., Budapest, Hungary) with Carl Zeiss objectives up to 41x bright field magnification by default. In the used bottom-up approach, the whole sample is acquired at high resolution. Low magnification representations are automatically obtained. Via Pannoramic Viewer 1.15.4 (open source software, 3D HISTECH Ltd., Budapest, Hungary) slides were evaluated and three captures with a magnification of × 200 were extracted of each sample.

The histopathological images were further analyzed by using the ImageJ software 1.48v (National Institutes of Health Image program) with a Windows operating system. Finally, expression of EGFR, VEGF, Hif1-alpha, and p53 (Figures 1C–G) was estimated as a sum of stained areas (μm^2^).

### Statistical Analysis

Statistical analysis was performed using SPSS package (IBM SPSS Statistics for Windows, version 22.0, Armonk, NY: IBM corporation). Collected data were evaluated by means of descriptive statistics.

Spearman's correlation coefficient (*ρ*) was used to analyze associations between investigated parameters. The Benjamini-Hochberg correction was used to adjust for multiple tests. Mann-Whitney-Test was used to test between groups. *P* < 0.05 were taken to indicate statistical significance.

## Results

**Figure 2:**
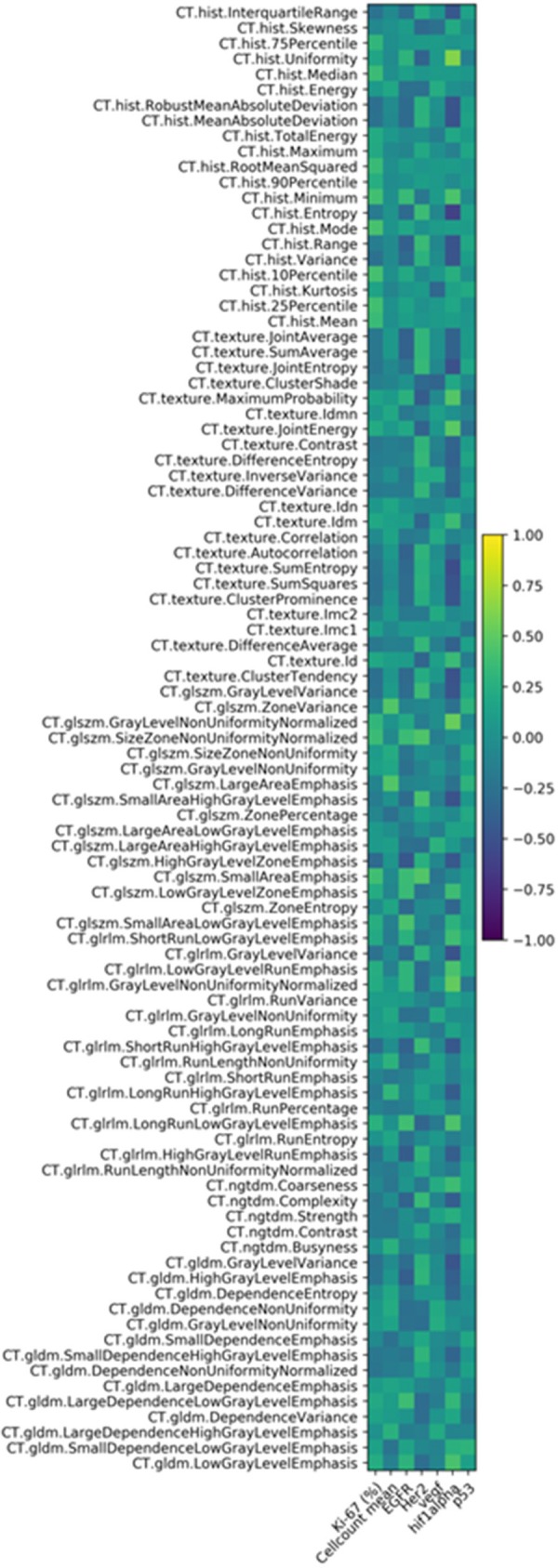
Correlation heat map of the investigated CT texture features and histopathology parameters.

**Figure 3:**
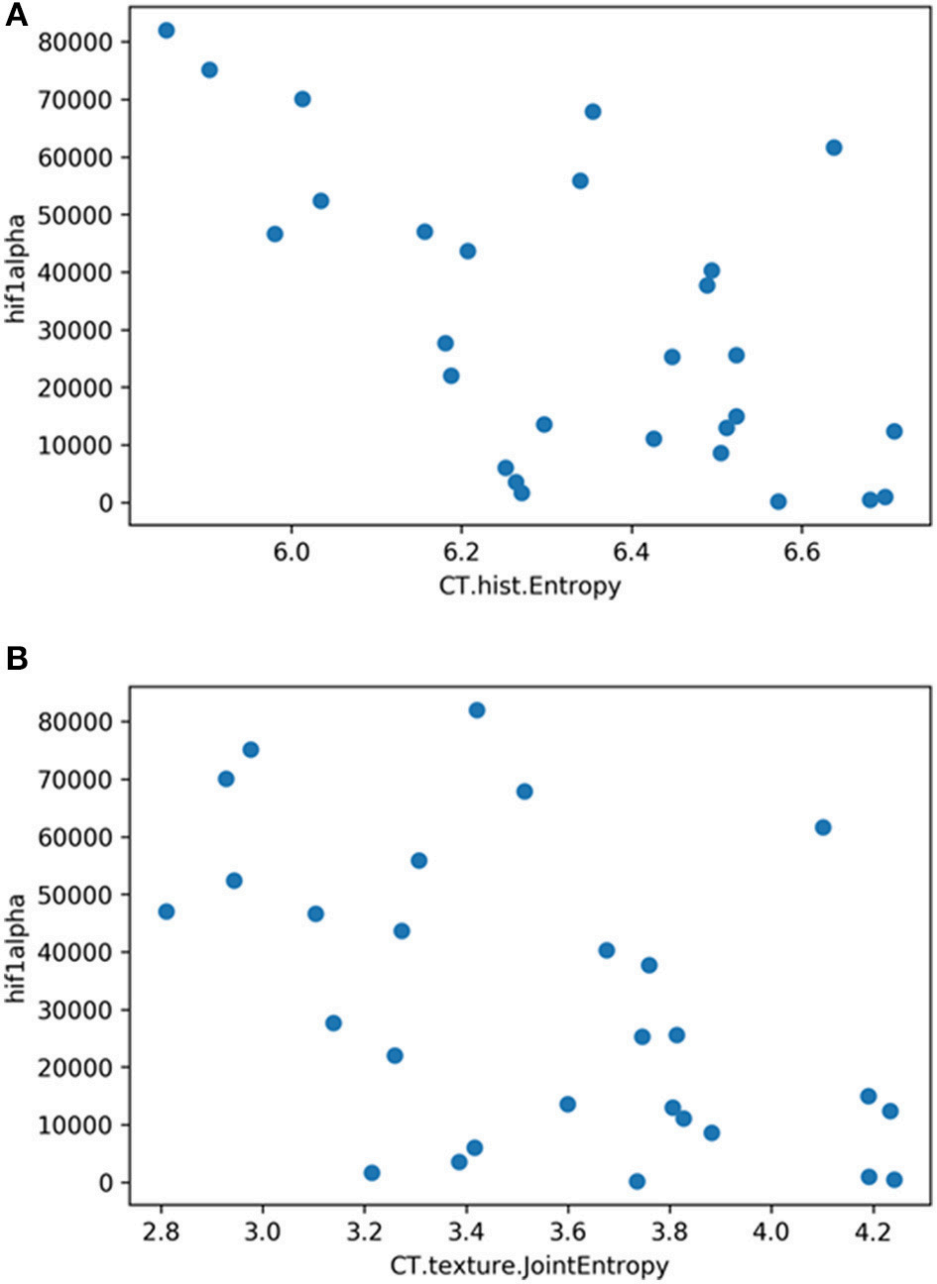
**(A)** Correlation graph showing the correlation between texture feature histogram entropy and Hif1-alpha expression. The correlation coefficient is *ρ* = −0.60. **(B)** Correlation graph showing the correlation between texture feature texture joint entropy and Hif1-alpha expression. The correlation coefficient is *ρ* = −0.50.

The correlation heat map summarizes the correlations between CT features and the histopathology parameters (Figure 2). Table 1 gives an overview about the highest correlations before correction for multiple tests. After Benjamini-Hochberg correction for multiple tests only 2 correlations remained statistically significant between CT texture joint entropy and CT entropy with Hif1-alpha expression (Figures 3A,B).

**Table 1:** Overview about the CT derived texture features and their correlations with histopathology.

**Texture feature**	**Histopathology parameter**	***r***	***P***
CT.hist.Uniformity	hif1alpha	0.631956	0.000309
CT.hist.Entropy	hif1alpha	−0.60379	0.000669
CT.glszm.GrayLevelNonUniformityNormalized	hif1alpha	0.532074	0.003565
CT.texture.SumEntropy	hif1alpha	−0.52926	0.003779
CT.glrlm.GrayLevelNonUniformityNormalized	hif1alpha	0.516409	0.004903
CT.texture.JointEntropy	hif1alpha	−0.50271	0.006402
CT.texture.ClusterTendency	hif1alpha	−0.49153	0.007898
CT.texture.JointEnergy	hif1alpha	0.49115	0.007954
CT.glszm.SmallAreaHighGrayLevelEmphasis	hif1alpha	−0.49102	0.007973
CT.texture.SumSquares	hif1alpha	−0.48339	0.009162
CT.hist.Range	hif1alpha	−0.4819	0.009413
CT.glrlm.GrayLevelVariance	hif1alpha	−0.47882	0.009945
CT.hist.MeanAbsoluteDeviation	hif1alpha	−0.47844	0.010012
CT.gldm.GrayLevelVariance	hif1alpha	−0.47413	0.010806
CT.glszm.GrayLevelVariance	hif1alpha	−0.47256	0.011106
CT.hist.Variance	hif1alpha	−0.47166	0.011282
CT.hist.RobustMeanAbsoluteDeviation	hif1alpha	−0.47082	0.011448
CT.texture.MaximumProbability	hif1alpha	0.466484	0.01234
CT.texture.ClusterProminence	hif1alpha	−0.46444	0.01278
CT.glszm.LargeAreaEmphasis	Cellcount	0.459229	0.018275
CT.glszm.ZoneVariance	Cellcount	0.459184	0.018288
CT.glszm.SmallAreaEmphasis	Her2	0.458725	0.016099
CT.glszm.SizeZoneNonUniformityNormalized	Her2	0.451696	0.018022
CT.glrlm.ShortRunHighGrayLevelEmphasis	hif1alpha	−0.44821	0.016755
CT.hist.InterquartileRange	hif1alpha	−0.4464	0.017257
CT.glszm.HighGrayLevelZoneEmphasis	EGFR	−0.446	0.017368
CT.glrlm.LongRunLowGrayLevelEmphasis	hif1alpha	0.444883	0.020061
CT.glszm.HighGrayLevelZoneEmphasis	hif1alpha	−0.44038	0.019012
CT.hist.Minimum	hif1alpha	0.43377	0.021103
CT.glszm.SmallAreaLowGrayLevelEmphasis	EGFR	0.42775	0.02317
CT.gldm.LowGrayLevelEmphasis	hif1alpha	0.425797	0.023875
CT.glrlm.LowGrayLevelRunEmphasis	hif1alpha	0.422941	0.024937
CT.glszm.SmallAreaHighGrayLevelEmphasis	Her2	0.420125	0.029122
CT.hist.Mode	Ki-67 (%)	0.418284	0.026752
CT.texture.MaximumProbability	Her2	−0.414	0.031808
CT.hist.10Percentile	Ki-67 (%)	0.41008	0.030208
CT.glrlm.HighGrayLevelRunEmphasis	EGFR	−0.4085	0.030913
CT.glrlm.LongRunHighGrayLevelEmphasis	hif1alpha	−0.40649	0.039331
CT.ngtdm.Complexity	hif1alpha	−0.40095	0.034471

*Spearman's rank coefficient were used. The p-values were not corrected for multiple tests*.

## Discussion

To the best of our knowledge this is the first study to analyze associations between CT texture features and histopathology in HNSCC. As shown, 2 CT texture features correlated with Hif1-alpha expression and, therefore, might be a surrogate marker for this histopathological parameter.

Previously, there are various studies elucidating the clinical benefit for texture and radiomics analyses in HNSCC (13, 19–22). For instance, Aerts et al. investigated 1,019 lung cancers and head and neck cancer patients with an extensive radiomics approach and could identify a radiomics signature, which was able to predict prognosis in both tumor entities (13). However, it was not able to predict human papilloma virus status, one of the most important prognostic markers in HNSCC (13). In another study, 149 HNSCC patients were investigated with a radiomics approach based upon contrast enhanced CT (20). A texture signature including 3 texture features, was able to predict local tumor control after definitive radiotherapy and HPV status (20). These results indicate that CT texture analysis may reflect relevant tumor microstructure in HNSCC.

Moreover, texture analysis of MRI images was also performed previously. So Scalco et al. combined the radiotherapy planning CT without contrast media application with conventional MRI sequences and DWI derived features to predict treatment response to chemo-radiotherapy in 30 patients (19). The CT features were not associated with treatment response, whereas features derived from t2-weighted images achieved an accuracy of 81.8% (19). Furthermore, by applying texture analysis on functional imaging modalities, like dynamic-contrast enhanced MRI, a study found out that the texture feature energy changed significantly during radiotherapy (22).

Despite its recent popularity among researchers in every field of medical imaging, there are still a there are still issues that surround the use of radiomics. Firstly, no study investigated possible underlying histopathological parameters that influence texture features and it can only be assumed that tumor heterogeneity might be linked to texture heterogeneity of analyzed images. There were only studies without a radiomics approach that identified that apparent diffusion coefficient (ADC)- values derived from Diffusions-weighted imaging (DWI) inversely correlated with cellularity and nucleic areas in HNSCC (4, 23, 24). Moreover, ADC was also associated with Ki67, a clinically most used proliferation index (3, 4).

Secondly, it is a concern that radiomics analyses lack standardization and might not be reliable enough for every day clinical practice (21, 25). In a recent study this problem regarding reliability of texture features was issued in lung cancer, HNSCC, and malignant mesothelioma (21). In fact, it has been identified that texture features in lung cancer showed the highest reliability, followed by HNSCC and malignant mesothelioma. Regarding HNSCC, the retrieved features were classified as stable and they are suitable for routine use, which strengthens our presented results.

In the present study, we identified an inverse correlation between several texture features and Hif1-alpha expression. This finding is very interesting and may be significant for clinical practice. In fact, Hif1-alpha plays a major role in supporting tumor metabolism and in cellular adaptation to hypoxic stress (26). Furthermore, it is associated with prognosis after radiotherapy (27). Our finding is somewhat interesting because a recent study identified no correlations between ADC values derived from DWI and Hif1-alpha expression (28). Presumably, texture analysis of CT images might, therefore, better reflect hypoxic areas in HNSCC and might be a very promising novel biomarker. It is known that entropy is the texture feature, which quantifies heterogeneity. The hypoxic microenvironment is highly dynamic and contains subpopulations of tumor cells exposed to changing gradients of oxygen (29). Presumably, this might be reflected by entropy derived from CT images.

The feature “glszm.SmallAreaEmphasis” was correlated with Her 2-expression, yet didn't reach statistical significance after correction for multiple tests. High expression of Her2 has been associated with tumor cell resistance to chemotherapy and radiotherapy (30). Consecutively, Her 2-overexpression was associated with disease free survival in a multivariate analysis (31). To the best of our knowledge no other imaging study to date was published to elucidate possible associations between CT texture features and Her 2- expression.

Furthermore, another texture feature “Glszm.HighGrayLevelZoneEmphasis” was the best parameter correlating with EGFR expression, but it also didn't reach statistically significance after correction. EGFR is involved in pathways related to the tumor microenvironment, tumor cell metabolism, and controls cell survival mechanisms such as proliferation, hypoxia resistance, DNA damage repair and apoptosis (32).

No texture parameter correlated with cell count or nucleic areas. It is widely acknowledged that ADC values derived from DWI might be capable to reflect cellularity in tumors (33). The present study shows that texture analysis derived from CT images might not be sensitive enough to reflect such microstructure changes in tumors.

There are several limitations of the present study to address. Firstly, it uses a retrospective design with possible known bias. However, to reduce this bias the evaluation of imaging and histopathology was conducted blinded and independently to each other. Secondly, our patient sample is relatively small. Thirdly, a known issue of clinical studies using a radiomics approach is the large number of generated features in relation to the acquired patient sample. Hence, *p*-values were corrected for multiple testing. Moreover, the texture analysis used in the present study is performed as a whole lesion measurement. Especially, for small tumors a voxel by voxel analysis might be more beneficial to reflect tumor heterogeneity. Another limitation is that we did not perform fractal or wavelet analyses which could better reflect geometrical complexity and thus might be also correlated with histopathological microstructure (34, 35). Presumably, further studies are needed to confirm our findings in other tumor entities and with a more advanced texture analysis approach.

In conclusion, CT texture analysis parameters might be associated with Hif1-alpha expression in HNSCC and therefore may be a promising novel biomarker in HNSCC.

## Ethics Statement

This study was carried out in accordance with the recommendations of the University of Leipzig Institutional Review Board, with written informed consent from all subjects. All subjects provided a written informed consent for their participation in this study and for their personal information to be used for research and publication. Written informed consent was obtained in accordance with the Declaration of Helsinki.

## Author Contributions

HM: data analysis and interpretation, manuscript writing, accountable for all aspects of the work, and manuscript approval. GH: CT data analysis and interpretation, statistical analysis, manuscript approval. AH: histopathology data analysis and interpretation, manuscript approval. AS: manuscript writing, manuscript approval, integrity of the work, study design.

### Conflict of Interest Statement

The authors declare that the research was conducted in the absence of any commercial or financial relationships that could be construed as a potential conflict of interest.
